# Development and Validation of a Stability Indicating RP-UPLC Method for Determination of Quetiapine in Pharmaceutical Dosage Form

**DOI:** 10.3797/scipharm.1009-12

**Published:** 2011-01-16

**Authors:** Rakshit Kanubhai Trivedi, Mukesh C. Patel

**Affiliations:** 1 Analytical Research and Development, Integrated Product Development, Dr. Reddy’s Laboratories Ltd., Bachupally, Hyderabad-500 072, India; 2 P. S. Science and H. D. Patel Arts College, S. V. Campus, Kadi-382 715, Gujarat, India

**Keywords:** Seroquel, Assay, Method validation, Degradation products, Rapid, Chromatography

## Abstract

The present work reports a stability indicating reversed phase ultra performance liquid chromatography (RP-UPLC) method for the quantitative determination of quetiapine in pharmaceutical dosage form. The chromatographic separation is performed on an Agilent Eclipse Plus C18, RRHD 1.8 μm (50 mm x 2.1 mm) column using gradient elution. The optimized mobile phase consists of 0.1 % aqueous triethylamine (pH 7.2) as a solvent-A and 80:20 v/v mixture of acetonitrile and methanol as solvent-B. The eluted compounds are monitored at 252 nm wavelength using a UV detector. The developed method separates quetiapine from its five impurities/degradation products within a run time of 5 min. Stability indicating capability of the developed method is established by analyzing forced degradation samples in which the spectral purity of quetiapine is ascertained along with the separation of degradation products from analyte peak. The developed RP-UPLC method is validated as per International Conference on Harmonization (ICH) guidelines with respect to system suitability, specificity, precision, accuracy, linearity, robustness and filter compatibility.

## Introduction

Quetiapine, {2-[4-(dibenzo[*b*,*f*][1,4]thiazepin-11-yl)piperazin-1-yl]ethoxy}ethanol, is an atypical antipsychotic drug with a unique receptor-binding profile belonging to a new chemical class, the dibenzothiazepine derivatives [1–4]. Quetiapine is an antagonist at a broad range of neurotransmitter receptors [3, 4]. Quetiapine is used in the treatment of schizophrenia or manic episodes associated with bipolar disorder. These antipsychotics have a low incidence of extrapyramidal side effects and tardive dyskinesias compared to older antipsychotics. Quetiapine (Quetiapine Fumarate, Seroquel™) is approved by the US Food and Drug Administration (FDA) in 1997. The advantages of the therapeutic profile of quetiapine have led to increasing use in the clinical practice, which encourages the development of new analytical method to provide driving force in today’s pharmaceutical industry. Higher sample throughput with more information per sample may decrease the time to market, an important driving force in today’s pharmaceutical industry. UPLC is a new category of separation technique based upon well-established principles of liquid chromatography, which utilizes sub-2 μm particles for stationary phase. These particles operate at elevated mobile phase linear velocities to affect dramatic increase in resolution, sensitivity and speed of analysis. Owing to its speed and sensitivity, this technique is gaining considerable attention in recent years for pharmaceuticals and biomedical analysis. In the present work, this technology has been applied to the method development and validation study of assay determination of quetiapine dosage forms.

**Fig. 1.**
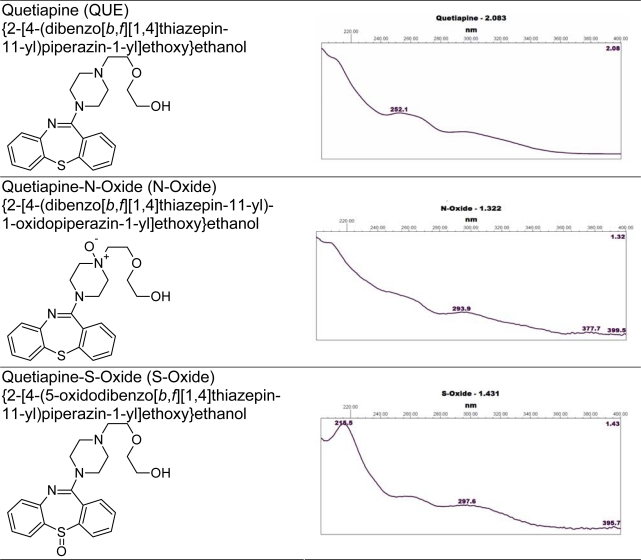

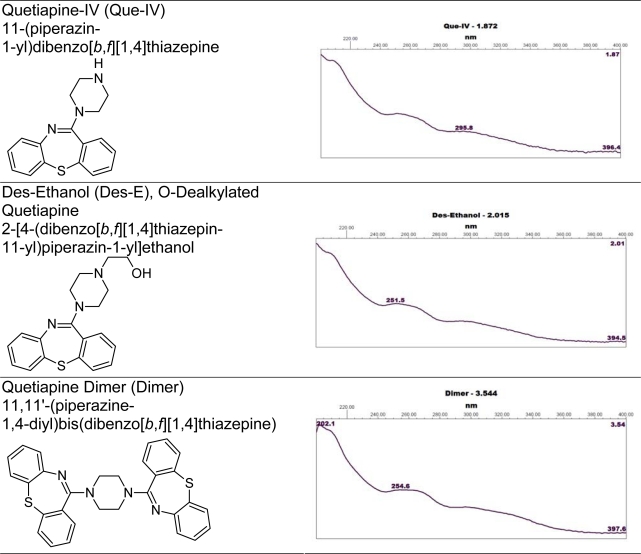
Chemical structures, IUPAC name and UV spectrums of quetiapine / impurities / degradation products

Also, the parent drug stability test guideline Q1A (R2) issued by the International Conference on Harmonization (ICH) [5] suggests that stress studies should be carried out on a drug to establish its inherent stability characteristics, leading to identification of degradation products and, hence, supporting the suitability of the proposed analytical procedures. It also requires that analytical procedures for testing the stability of samples should be stability-indicating and should be fully validated. Chemical structures, IUPAC name and UV spectrums of quetiapine / impurities / degradation products were presented in Figure 1.

A detailed literature survey for quetiapine revealed that few analytical methods are available using HPLC were; S. Radha Krishna *et al*. [6], describe stability indicating method for related substances of quetiapine drug substance; F. Belal *et al*. [7], demonstrated separation of two impurity from quetiapine; V. Pucci et al. [8], determination of quetiapine was describe with non stability indicating method. Polarographic method is also reported for the analysis of quetiapine in pharmaceuticals [9]. HPTLC method is also reported by S.R. Dhaneshwar *et al*. [10]. Determination of quetiapine by Capillary zone electrophoretic method is reported by S. Hillaert *et al.* [11]. A Voltammetric analysis of quetiapine in human serum and urine is also reported [12]. GC/MSD [13], LC-MS [14–22], and LC-MS-MS [23] are reported for determination of quetiapine in drug substance, pharmaceutical formulations and biological matrices. Identification and characterization of quetiapine impurities was also reported [24, 25].

To the best of our knowledge, none of the currently available analytical methods can separate all the known related compounds and degradation impurities of quetiapine dosage form. Quetiapine pharmaceutical formulation is also not official in any pharmacopoeia yet. Furthermore, there is no less time-consuming and stability-indicating RP-UPLC method reported in the literature that can adequately separate all the substance and accurately quantify quetiapine dosage form. Also the cost of the analysis using LC-MS, GC/MSD and LC-MS-MS is very high and also very delicate instrument are needed compared to UPLC with respect to routine quality control analysis. Hence, we focused on developing a selective, fast, cost-effective and stability-indicating method using this advance technique (UPLC) for the assay determination of quetiapine in solid pharmaceutical dosage forms.

Hence a reproducible stability-indicating RP-UPLC method was developed which is less time-consuming and more selective compared to the all present methods, which takes only about 5 min for a single run. Thereafter, this method was successfully validated according to the ICH guideline [27].

## Results and Discussion

### Method development and optimization

The main criteria for development of successful UPLC method for determination of QUE in tablets were: the method should be able to determine assay of drug in single run and should be accurate, reproducible, robust, stability indicating, free of interference from blank / placebo / impurities / degradation products and straightforward enough for routine use in quality control laboratory.

The spiked solution of related compounds (N-Oxide, S-Oxide, Que-IV, Des-E, Dimer) and QUE were subjected to separation by RP-UPLC. The Rasayan Journal of Chemistry [6] literature for QUE states a RP-HPLC method using C18 column for its related substances. The mobile phase was containing a gradient mixture of Solvent-A (5mM of ammonium acetate) and Solvent-B (acetonitrile) [6]. The separation of all compounds form QUE was studied using this composition on UPLC column (Acquity BEH C18 50 x 2.1 mm, 1.7μm) and Waters (UPLC) system with the linear gradient program (Table 1). The flow rate of 0.5 mL/min was selected with regards to the backpressure and analysis time as well. When studied was performed with above condition we observed that N-Oxide and S-Oxide was co-eluting and Des-E and QUE were found co-eluting next to them. Que-IV is well separated from QUE and Dimer was relatively retained for longer time and it was well separated from peak of QUE. Based on this experiment Des-E and QUE was selected as a critical pair for separation. During this study column oven temperature was capped 40°C. Various types of solvents A and B were studied to optimize the method, which were summarized in Table 2 with the observation. Based on above solvent selection experimental study optimized UPLC parameters were; flow rate 0.5mL/min; column oven temperature 40°C; gradient solvent program as per Table 1; as a Solvent-A 0.1 % aqueous triethylamine (adjusted to pH 7.2 with orthophosphoric acid) and as Solvent-B mixture of acetonitrile and methanol in the ratio of 80:20 (v/v) respectively.

**Fig. 2.**
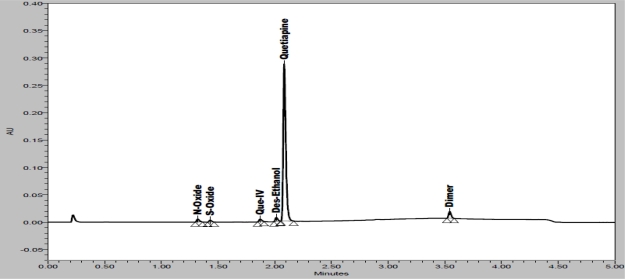
Spiked chromatogram of QUE along with impurities

**Fig. 3.**
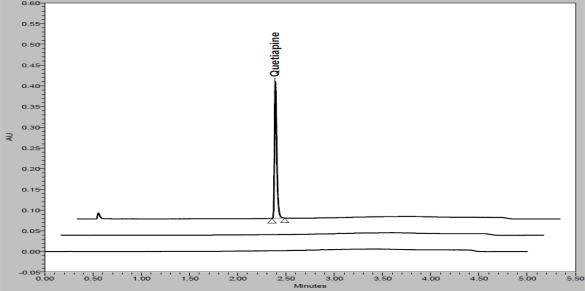
Overlay chromatograms of blank (bottom), placebo (middle) and standard (top) preparation

In order to achieve symmetrical peak of QUE and more resolution between Des-E and QUE, various stationary phases like C8 (different dimension), C18 (different brand), phenyl were also studied. Summary of stationary phases are presented in Table 3. Based on this summary it was concluded that Eclipse Plus C18; RRHD (50 x 2.1mm, 1.8μm) giving a better resolution (Des-E and QUE) and symmetrical peaks of all component with respect to other stationary phases and other equivalent columns. Column oven temperature is also studied (at room temperature and 40°C) and found that 40°C temperature is more appropriate with respect to resolution and peak shape. The wavelength of QUE maximum (252 nm) was used to have good detection of QUE and impurities (at 2.5 % of standard concentration). QUE and their impurities were well resolved in reasonable time of 5 minutes which was presented in Figure 2 (spiked chromatogram of QUE along with impurities). There was no any interference of excipients (placebo) and blank (diluent) at the retention time of QUE peak. Overlay chromatograms of blank, placebo and standard solution is presented in Figure 3.

### Analytical parameters and validation

After satisfactory development of method it was subjected to method validation as per ICH guideline [27]. The method was validated to demonstrate that it is suitable for its intended purpose by the standard procedure to evaluate adequate validation characteristics (system suitability, accuracy, precision, linearity, robustness, solution stability, filter compatibility and stability indicating capability).

### System suitability

System suitability parameters were measured so as to verify the system, method and column performance. The % RSD (relative standard deviation) of QUE area count of six replicate injections (standard preparation) was below 0.4 %. Low values of % RSD of replicate injections indicate that the system is precise. Results of other system suitability parameters such as resolution, theoretical plates, tailing factor, capacity factor and similarity factor (between two standard preparations) are presented in Table 4. As seen from this data, the acceptable system suitability parameters would be: resolution between Des-E and QUE is not less than 1.5, theoretical plates for QUE is not less than 30000, tailing factor for QUE is not more than 2.0, similarity factor (between two standard preparations) is not less than 0.98 and not more than 1.02 and % RSD of replicate injections is not more than 2.0 %. Results of system suitability parameters from different studies are also presented in Table 4. Spiked chromatogram of impurity / degradation products with QUE is presented in Figure 2.

### Specificity

Specificity is the ability of the method to measure the analyte response in the presence of its potential impurities [26]. Forced degradation studies were performed to demonstrate selectivity and stability indicating capability of the proposed RP-UPLC method. Figure 2 and 3 are shows that there is no any interferences at the RT (retention time) of QUE due to blank, placebo, impurities and degradation products. Significant degradation was not observed when QUE was subjected to acid, alkali, thermal, hydrolytic and UV conditions. Significant degradation was observed when the drug product was subjected to oxidative hydrolysis (30% H_2_O_2_, 60°C, 1h) leading to the formation of N-Oxide and S-Oxide. Overlay chromatograms (blank, placebo and sample) of peroxide degradation study are presented in Figure 4A along with QUE purity plot (Figure 4B). Peak due to QUE was investigated for spectral purity in the chromatogram of all exposed samples and found spectrally pure. The purity and assay of QUE was unaffected by the presence of its impurities and degradation products and thus confirms the stability-indicating power of the developed method. Results from forced degradation study are given in Table 5.

### Precision

The precision of the assay method was verified by repeatability and by intermediate precision. Precision was investigated using sample preparation procedure for six real samples of tablets and analyzing by proposed method. Intermediate precision was studied using different column, and performing the analysis on different day. The average % assay (n=6) of QUE was 99.9 with RSD of 0.38 %. Results are presented in Table 6 along with intermediate precision data. Low values of % RSD, indicates that the method is precise.

### Accuracy

To confirm the accuracy of the proposed method, recovery experiments were carried out by standard addition technique. Five different levels (50 %, 75 %, 100 %, 125 % and 150 %) of standards were added to pre-analyzed placebo samples in triplicate. The percentage recoveries of QUE at each level and each replicate were determined. The mean of percentage recoveries (n =15) and the % RSD was calculated. The amount recovered was within ±1 % of amount added, which indicates that the method is accurate and also there is no interference due to excipients present in tablets. The results of recoveries for assay are shown in Table 7.

### Linearity

**Fig. 5.**
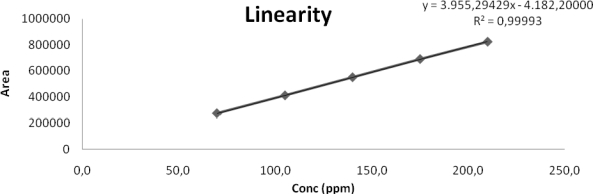
Linearity curve

Linearity was demonstrated from 50 % to 150 % of standard concentration using minimum five calibration levels (50 %, 75 %, 100 %, 125 % and 150 %) for the QUE compound, which gave us a good confidence on analytical method with respect to linear range. The response was found linear for QUE from 50 % to 150 % of standard concentration and correlation coefficient was also found greater than 0.9999. Y-intercept bias was also found within ± 2. The result of Correlation coefficients, Y-intercept bias and linearity equations for QUE are presented in Table 8. Linearity curve is presented in Figure 5.

### Robustness

The robustness as a measure of method capacity to remain unaffected by small, but deliberate changes in chromatographic conditions was studies by testing influence of small changes in flow rate (± 0.05 mL/min), change in column oven temperature (± 3°C) and change in Sol-B (± 10 % of methanol composition). No significant effect was observed on system suitability parameters such as resolution, theoretical plates, tailing factor, capacity factor, similarity factor and % RSD of QUE, when small but deliberate changes were made to chromatographic conditions. The results are presented in Table 4 along with system suitability parameters of precision and intermediate precision study. Thus, the method was found to be robust with respect to variability in above conditions.

### Stability of sample solution

Stability of sample solution was established by storage of sample solution at ambient temperature for 24 h. QUE sample solution was re-analyzed after 12 and 24 h time intervals and assay was determined and compared against fresh sample. Sample solution did not show any appreciable change in assay value when stored at ambient temperature up to 24 h, which are presented in Table 9. The results from solution stability experiments confirmed that sample solution was stable for up to 24 h during assay determination.

### Filter compatibility

Filter compatibility was performed for nylon 0.2 μm syringe filter (Pall Life sciences) and PVDF 0.2 μm syringe filter (Millipore). To confirm the filter compatibility in proposed method, filtration recovery experiment was carried out by sample filtration technique. Sample was filtered through both syringe filter and percentage assay was determined and compared against centrifuged sample. Sample solution was not showing any significant changes in assay percentage with respect to centrifuged sample. Percentage assay results are presented in Table 10. In displayed result difference in % assay was not observed more than ±0.2, which indicates that both syringe filters having a good compatibility with sample solution.

## Experimental

### Materials and Reagents

Tablets and standard of Quetiapine Fumarate (99.5 %) and its five impurities namely N-oxide (98.1 %), S-oxide (98.5 %), Que-IV (99.2 %), Des-ethanol (96.8 %) and Dimmer (98.4 %) were provided by Dr. Reddy’s laboratories Ltd., Hyderabad, India. HPLC grade acetonitrile and methanol were obtained from J.T.Baker (NJ., USA). GR grade orhtophosphoric acid and GR grade triethylamine were obtained from Merck Ltd. (Mumbai, India). 0.2 μm nylon membrane filter and nylon syringe filters were purchased from Pall life science limited (India). 0.2 μm PVDF syringe filter was purchased from Millipore (India). High purity water was generated by using Milli-Q Plus water purification system (Millipore, Milford, MA, USA).

### Equipments

Cintex digital water bath was used for specificity study. Photo stability studies were carried out in a photo-stability chamber (Sanyo, Leicestershire, UK). Thermal stability studies were performed in a dry air oven (Cintex, Mumbai, India).

### Chromatographic conditions

Analyses were performed on Acquity UPLC system (Waters, Milford, USA), consisting of a binary solvent manager, sample manager and PDA (photo diode array) detector. System control, data collection and data processing were accomplished using Waters Empower-2 chromatography data software. The chromatographic condition was optimized using Agilent Eclipse Plus C18, RRHD 1.8 μm (50 mm x 2.1 mm) column. 0.1% aqueous triethylamine (adjusted to pH 7.2 with orthophosphoric acid) was used as a solvent-A and mixture of acetonitrile and methanol in the ratio of 80:20 (v/v) respectively was used as solvent-B. Solvents-A and B was filtered through 0.2 μm nylon membrane filter and degassed under vacuum prior to use. The separation of QUE and impurities was achieved by gradient elution using Sol-A and Sol-B. Mixture of water, acetonitrile and perchloric acid in the ratio of 200:800:0.13 (v/v/v) respectively was used as a diluent. The finally selected and optimized conditions were as follows: injection volume 1 μL, gradient elution (Table 1), at a flow rate of 0.5 mL/min at 40°C (column oven) temperature, detection wavelength 252 nm. The stress degraded samples and the solution stability samples were analyzed using a PDA detector covering the range of 200–400nm.

### System suitability solution preparation

System suitability solution was prepared by dissolving standard substance and impurity in diluent to obtain solution containing 3 μg/mL of Des-E and 125 μg/mL of QUE.

### Standard solution preparation

Standard solution was prepared by dissolving standard substance in diluent to obtain solution containing 125 μg/mL of QUE.

### Sample solution preparation

Twenty tablets were crushed to fine powder. An accurately weighed portion of the powder equivalent to 25 mg of QUE was taken into 200 mL volumetric flask. About 150 mL of diluent was added to this volumetric flask and sonicated in an ultrasonic bath for 10 min. This solution was then diluted up to the mark with diluent and mixed well. It was then filtered through 0.2 μm PVDF syringe filter and the filtrate was collected after discarding first few milliliters.

## Conclusion

A gradient RP-UPLC method was successfully developed for the estimation of quetiapine in pharmaceutical dosage form. The method validation results have proved that the method is selective, precise, accurate, linear, robust, filter compatible and stability indicating. The run time (5.0 min) enables for rapid determination of drug. Moreover, it may be applied for determination of QUE in the study of blend uniformity, tablet content uniformity and *in-vitro* dissolution profiling of QUE dosage forms, where sample load is higher and high throughput is essential for faster delivery of results.

## Figures and Tables

**Fig. 4A.**
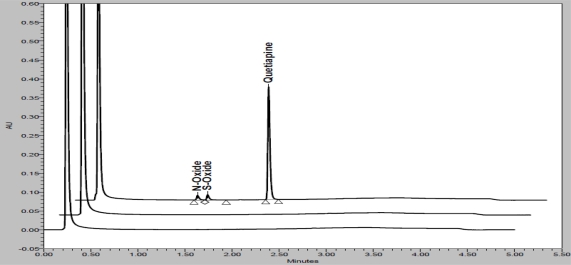
Overlay chromatograms of peroxide degradation study [blank (bottom), placebo (middle) and degraded sample (top)]

**Fig. 4B.**
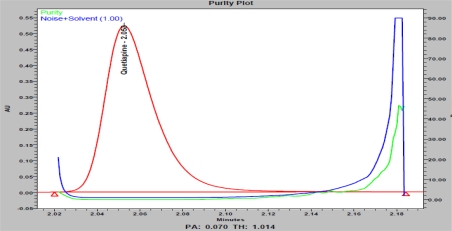
Quetiapine peroxide degraded sample purity plot

**Tab. 1.** Gradients program for elution

**Time (min)**	**Flow rate (mL/min)**	**% Solvent-A**	**% Solvent-B**	**Gradient curve**
Initial	0.5	70	30	Isocratic
0.5	0.5	70	30	Isocratic
3.0	0.5	5	95	Linear
4.0	0.5	5	95	Isocratic
4.1	0.5	70	30	Isocratic
5.0	0.5	70	30	Equilibration

**Tab. 2.** Summary of solvent used to optimize the method

**Solvent-A (Sol-A)**	**Solvent-B (Sol-B)**	**Observation/Remarks**
5 mM Ammonium acetate solution	Acetonitrile	Co-eluting peak of Des-E and QUE was observed
0.1 % aqueous triethylamine	Acetonitrile	Co-eluting peak of Des-E and QUE was observed
0.1 % aqueous triethylamine (adjusted to pH 7.2 with H_3_PO_4_)	Mixture of acetonitrile and water in the ratio of 90:10 (v/v)	Poor resolution between Des-E and QUE, and higher peak tailing for the peak of QUE were observed.
0.1 % aqueous triethylamine (adjusted to pH 7.2 with H_3_PO_4_)	Mixture of acetonitrile and methanol in the ratio of 80:20 (v/v)	1.3 USP resolution was observed between Des-E and QUE

**Tab. 3.** Summary of stationary phase used to optimize the method

**Stationary phase**	**Dimension**	**Observation/Remarks**
Acquity BEH C8	(50 x 2.1) mm, 1.7 μm	Poor resolution between Des-E and QUE
Acquity BEH C8	(100 x 2.1) mm, 1.7 μm	Poor resolution between Des-E and QUE
Acquity BEH Phenyl	(50 x 2.1) mm, 1.7 μm	Peak merging of Des-E and QUE
Acquity BEH C18	(50 x 2.1) mm, 1.7 μm	Poor resolution between Des-E and QUE
Eclipse Plus C18, RRHD	(50 x 2.1) mm, 1.8 μm	Satisfactory resolution between Des-E and QUE

**Tab. 4.** System suitability results (precision, intermediate precision and robustness)

**Parameter**	**Resolution between Des-E and QUE**	**Theoretical plates for QUE**	**Tailing factor for QUE**	**Capacity factor for QUE**	**Standard Similarity factor**	% RSD* of Standard Area
Precision	1.61	38771	1.50	6.95	1.01	0.31
Intermediate Precision	1.64	38213	1.48	6.97	0.99	0.27
At 0.45 mL/min flow rate	1.52	35191	1.55	7.27	0.99	0.35
At 0.55 mL/min flow rate	1.72	40663	1.44	6.65	1.00	0.21
At 37°C Column temp.	1.61	37611	1.50	6.94	1.01	0.28
At 43°C Column temp.	1.62	38781	1.47	6.91	1.00	0.20
Sol-B [−10 % methanol]	1.61	37807	1.47	6.92	0.99	0.34
Sol-B [+10 % methanol]	1.60	37972	1.48	6.90	1.01	0.22

* Determined on six values.

**Tab. 5.** Summary of forced degradation results

**Degradation condition**	**Assay (% w/w)**	**Purity Flag**	Major degradation products*
Control sample	99.9	No	N.A.
Acidic hydrolysis (1N HCl, 60°C, 1 h)	99.2	No	No significant degradation observed
Alkaline hydrolysis (1N NaOH, 60°C, 1 h)	98.8	No	No significant degradation observed
Oxidation (30% H_2_O_2_, 60°C, 1 h)	90.0	No	N-Oxide (4.25 %)* and S-Oxide (4.68 %)*
Water hydrolysis (60°C, 24 h)	99.5	No	No degradation observed
Thermal (60°C, 6 h) solid	100.1	No	No degradation observed
Exposed to UV at 254nm	100.3	No	No degradation observed

* Percentage area against quetiapine; N.A. Not applicable.

**Tab. 6.** Precision and Intermediate precision results

**Quetiapine**	**Precision at 100 %**		**Intermediate precision**	

	% Assay #	% RSD*	% Assay #	% RSD*

	99.9	0.38	100.2	0.39

# Average of six determinations;

* Determined on six values.

**Tab. 7.** Accuracy results

**Quetiapine**	**At 50 %**	**At 75 %**	**At 100 %**	**At 125 %**	**At 150 %**
% Recovery #	99.4	99.8	99.7	100.0	99.3
% R.S.D.*	0.40	0.35	0.25	0.26	0.20

* Determined on three values;

# Mean of three determinations.

**Tab. 8.** Linearity results

**Linearity range (μg/mL)**	**Correlation Coefficient (r^2^)**	**Linearity (Equation)**	**Y-intercept bias**
62.5 to 187.5	0.99993	y = 3955.2943(x) − 4182.2	−1.865

**Tab. 9.** Solution stability results

**% Assay**	**Initial**	**After 12 hrs.**	**After 24 hrs.**

	100.1	99.9	99.8

**Tab. 10.** Filter compatibility results

**% Assay**	**Centrifuged**	**PVDF Syringe filter 0.2μm (Millipore)**	**Nylon Syringe filter 0.2μm (Pall Life Sciences)**

	99.9	100.1	99.8
